# An AI-driven framework for evaluating local and state authorities’ permitting processes

**DOI:** 10.1038/s41598-026-53770-3

**Published:** 2026-06-16

**Authors:** Ranjit R. Desai, Umapriya Renganathan, Reid Olson, Erin Andrews-Sharer, Noah Frey, Alexandria Danner, Grant Buster, Jack Titus, Noel Crisostomo

**Affiliations:** 1 Center for Integrated Mobility Sciences, National Laboratory of the Rockies, 15013 Denver West Parkway, Golden, CO USA; 2 Strategic Energy Analysis Center, National Laboratory of the Rockies, 15013 Denver West Parkway, Golden, CO USA; 3 Office of Policy, United States Department of Energy, 1000 Independence Avenue, Washington, DC USA

**Keywords:** Permitting process, Soft costs, Infrastructure, Large language models, Generalized permitting process, Authorities having jurisdiction, Energy and society, Energy science and technology, Engineering, Mathematics and computing

## Abstract

The demand for new energy infrastructure is increasing across the United States, but heterogenous permitting processes and embedded requirements across different local jurisdictions can cause project delays, increase “soft costs,” and hinder developer expansion. This study analyzes the variability in local permitting requirements across the U.S. and develops a quantitative approach to describe their clarity and effectiveness in enabling infrastructure project development. By using an Energy Language Model (ELM), a large language model (LLM) for energy technologies, we systematically gathered permitting information from nearly 300 state-, county-, and city-level documents, creating a structured dataset of requirements and procedures on an unprecedented scale and speed. Our analysis revealed that local (city and county) permitting requirement documents are underrepresented compared to state-level guidance documents, which can impede timely and cost-effective installation of new electric infrastructure. Our validation process showed that the final database has an accuracy of approximately 95%. We, further, created a new quantitative method to score permitting requirements for clarity and efficiency, with electric vehicle supply equipment as an initial use case. The average local permitting document scored a 1.8 out of 5, which we interpret as meaning that half of the requirements developers face when installing electric infrastructure are ambiguous, increasing both cost and time. We also created a “Generalized Permit Process”, highlighting common procedural steps and identifying specific opportunities for municipalities to improve their documentation. This research establishes a systematic and scalable framework for evaluating the complexities of local infrastructure permitting processes by combining LLM-powered data collection and quantitative scoring. The framework enables policymakers and developers to identify and mitigate procedural bottlenecks, with the expectation that these improvements can accelerate application review and approval, reduce project costs, and expedite connection to utility distribution grids. As a foundational approach for streamlining local project development processes, this study’s methods are intended to be extended to a wide range of energy applications.

**Supplementary Information:**

The online version contains supplementary material available at https://doi.org/10.1038/s41598-026-53770-3.

## Introduction

The pressing need for new electricity infrastructure in the United States (U.S.) is accelerating as demand is expected to accelerate from a 0.5% compound annual growth from 2014 to 2024 to 2.5% per year in 2025–2035^1^. For example, electricity demand solely from data centers is expected to cross 100 gigawatts by 2035^2,3^. Many studies have asserted that permitting reforms are critical to meet this increasing demand^1,4–9^. The U.S. Department of Energy (U.S. DOE) devised that artificial intelligence (AI) could aid in resolving the permitting and siting issues of deploying energy technologies, and launched an initiative for the *“Safe, Secure, and Trustworthy Development and Use of AI”*^10^. A key goal for AI-powered tools is to accelerate the siting and permitting process, including through comment processing, information extraction, document drafting, compliance check automation, and others^11,12^. The National Laboratory of the Rockies (NLR, previously NREL) created the Energy Language Model (ELM), a large language model (LLM) to support project development for electric generators. This study builds upon the ELM^13^ and methods documented in Buster et al.^14^ with AI enhancements in order to, as a novel contribution to the literature, analyze the local permitting workflows for new loads connecting to electric distribution systems.

This study has three objectives: first, to furnish a national assessment of existing permitting procedures governed by authorities having jurisdiction (AHJ) at various levels to formulate a “*Generalized Permitting Process”* that encapsulates a typical procedure for developers installing new load. Second, to quantify the variability in codes and requirements, leveraging LLMs to construct a structured database of permitting procedures. Third, to develop a novel scoring method for assessing the “wellness” of a permitting process, which allows for an evaluation of the process’s definitional clarity.

To achieve these objectives, we employed NLR’s ELM to search for and automate the extraction of key data requested during the permitting of charging infrastructure from hundreds of legal documents spanning state-, county-, and city-level AHJs. The extracted elements from these permitting documents were collected into a structured database, which allowed us to organize and condense varied jurisdictional requirements into the Generalized Permitting Process. Next, we interviewed industry stakeholders on their experiences in following permitting requirements, and how specific jurisdictions’ processes vary in strength or weakness. These evaluations revealed options to streamline local permitting processes by replacing ambiguous requirements with enhancements that could offer applicants opportunities to self-affirm that their project at issue is designed to relevant and specific criteria. By combining successful practices from the structured database, we developed a scoring method to evaluate the clarity of hundreds of individual AHJ’s procedures. Analysis of the scores identifies opportunities to improve permitting processes and avoid key omitted elements, and the key omitted elements that may increase a project’s timeline and costs.

The costs to permit installations of public electric vehicle supply equipment to utility distribution systems have been the subject of recent studies^15,16^ following the introduction of new options of light duty passenger vehicles in the U.S.^17,18^. In early 2026, the U.S. had nearly 240,000 public Level 2 and DC fast charging plugs^19^. Addition of this public infrastructure may enable longer-distance travel for existing drivers and may make electric vehicles (EVs) usable for a broader range of the population^17,20–25^. However, charging infrastructure developers face inconsistency in permitting processes across multiple AHJs that oversee local codes and regulations^24^. The variable requirements to obtain permits reduces certainty in the timeline and cost of installations^15,26–28^. These delays and non-hardware costs add to the “soft costs” of charging infrastructure^15,16,26,29–31^.

Most existing permitting research describes best practices and associated policy options. However, there is limited quantitative investigation into how various codes (i.e., electrical, zoning, building, etc.) that are required to complete charging infrastructure projects are implemented across jurisdictions nationwide. Developers seeking to install a charging station must apply to their AHJs, who are responsible for verifying that the installation complies with all relevant national and international codes^24^. Project type dictates permit application details, including the need for utility grid connections to supply the station with electricity. Local zoning laws also control building types, locations, and land use^17,24,32,33^. A charging station may be subject to supplemental building and zoning codes, depending upon its structure and location^17,24^.

The effort developers must expend to comply with varied codes across AHJs is often exacerbated by the absence of detailed municipal code provisions and the insufficient knowledge about emerging EV technology among AHJ staff responsible for evaluating permit applications^15,17,24,26,29,34^. Ambiguous codes lead to uncertainty among AHJs and applicants regarding EV charging regulations, permits, and designated locations^17,24,34–36^. These code variations also introduce variability in the permitting process^17,24,34–36^. To illustrate, in 2019, charging projects in the U.S. required an average of 1.86 design revisions, while projects in California required 2.41 design revisions^15^. Further, ambiguity in the codes and subsequent code revisions can prolong the timeframe necessary for a project to complete permitting.

While the challenges of permitting are widely acknowledged, there has been no systematic, data-driven method to compare the complexity of codes across jurisdictions. This study addresses this gap by introducing a novel framework for analyzing and scoring permitting processes. Specifically, we: (1) apply a Large Language Model in a first-of-its-kind application to parse complex regulatory text into a structured database, quantifying permitting variability at scale; and (2) develop and validate a novel scoring metric to objectively assess the clarity and effectiveness of permitting requirements. This manuscript is structured with a methods section describing model implementation, an analysis of the structured database^35^ and the scoring method, a discussion of results, and conclusion.

## Methods

**Fig. 1:**
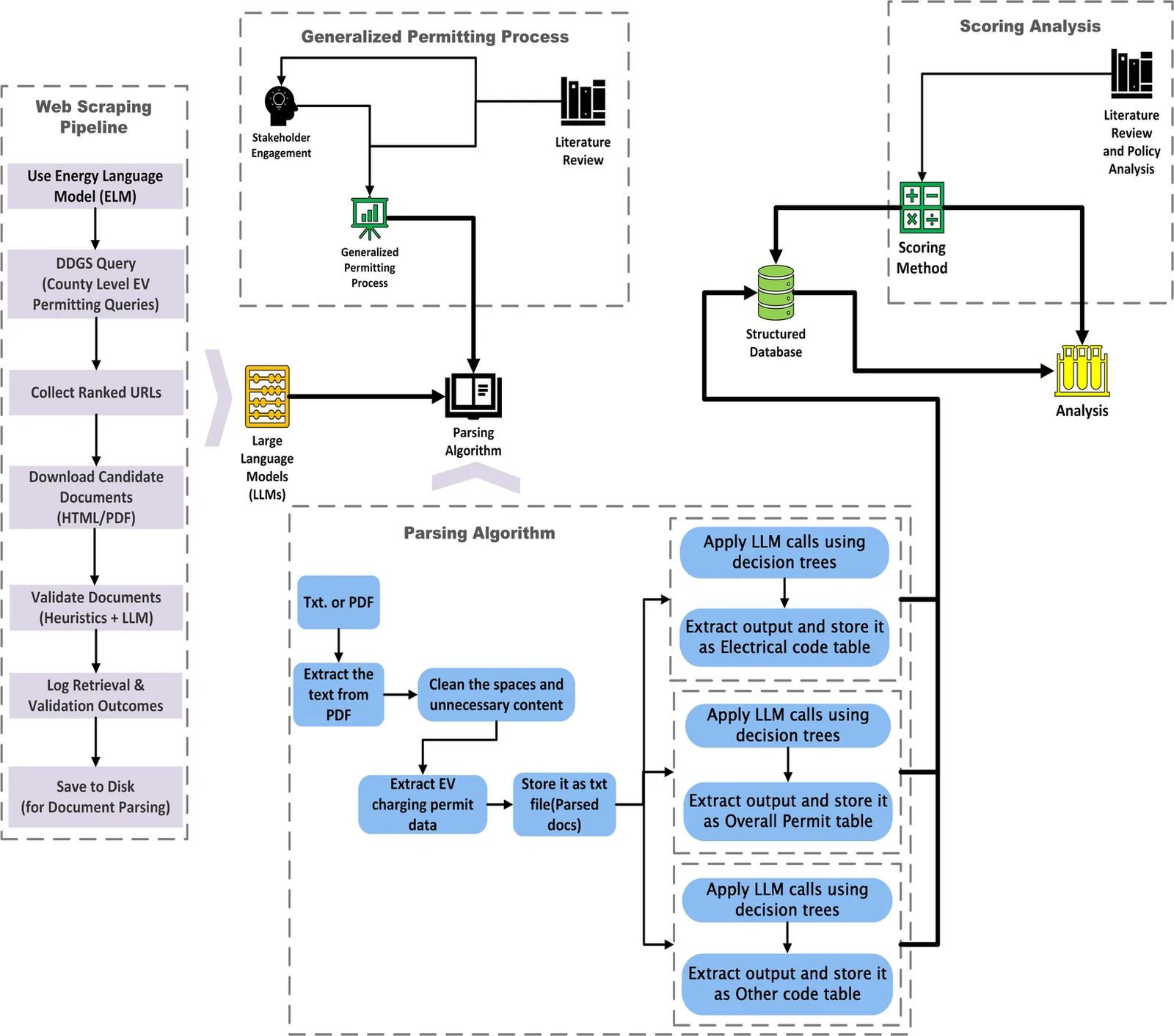
The analysis method consists of four components. First, the **Generalized Permitting Process** synthesizes stakeholder engagement and literature review into a workflow representing a typical Path to Approval. Second, the **Web Scraping Pipeline** uses NLR’s Energy Language Model (ELM)^13,37^ to download permitting documents. Third, the **Parsing Algorithm** processes these documents via ELM and integrates them with the generalized process to produce a Structured Database of EV permitting processes, available through the Open Energy Data Initiative^35^. Finally, a **Scoring Method** applies to evaluate the permitting processes.

Figure 1 illustrates the architecture employed in this study. The first component focuses on the formation of the Generalized Permitting Process. The generalized permitting process shows a typical pathway for the approval of permit applications for the installation of non-residential EV charging stations. This task included a comprehensive review of existing permitting processes to analyze the usual workflow in a typical permitting process across AHJs, and the discussions and opinions from specialists and stakeholders. The second stage of this project includes the use of LLM to obtain permitting processes from AHJs, and parsing these documents into a structured database. Lastly, we developed a novel scoring method by synthesizing the best practices and published policy analyses to analyze the permitting processes in the database.

### Generalized permitting process

**Fig. 2:**
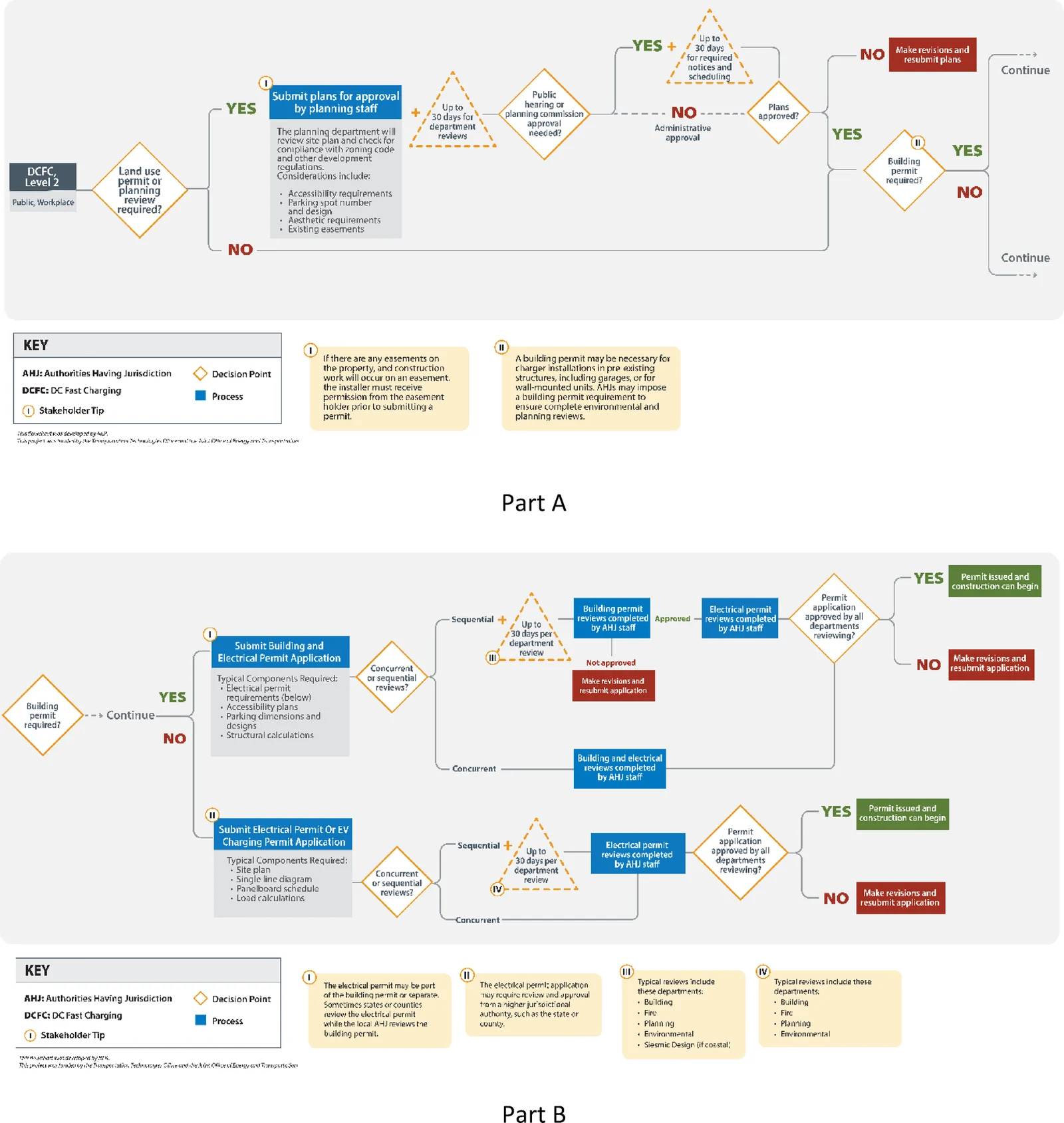
Generalized Permitting Process, Part B. Permitting Process is a key process in the EV charging installation process. The process includes Project Initiation, Engineering and Design, Contract and Payment, Easements and Permitting, Construction, and Energization^38^. This workflow is a typical” path to approval” of a permitting application to install non-residential EV charging stations and shows the essential stages in a typical EV permitting process. The first stage of the process illustrates the steps involved if a land use or zoning permit, or plan review is required. AHJs often have different terminology for this component of the process, which may be a preliminary step prior to submitting the other required permits. The workflow uses the “Stakeholder Tips” to provide additional details and recommend a list of documents or highlight a list of components from the application that would affect the particular stage. Further, the workflow features processes that can be concurrent or sequential depending on the respective AHJ. Additionally, the workflow shows whether a delay may occur if there is a rejection or denial at a particular step.

We employed a mixed-methods approach to develop a Generalized Permitting Process for EV charging station installation. Figure 1 shows the detailed method for this paper, and Fig. 2A & B shows the completed Generalized Permitting Process. The process comprised the following key stages, as shown in Figs. 1 and 2:

- **Document review:** A comprehensive review of policy documents from municipalities, counties, states, and federal entities was conducted to identify sequential steps for obtaining permits. Data collected from these documents facilitated the identification of common elements in application processes across different authorities having jurisdiction (AHJs), which were synthesized into a preliminary model.
- **Stakeholder engagement:** Semi-structured expert interviews were conducted with stakeholders actively involved in EV charging station installation, including manufacturers, installers, and city officials. These interviews aimed to validate the preliminary permitting model and acquire a comprehensive understanding of permitting requirements across various jurisdictions.
- **Qualitative data analysis:** We analyzed the information received during the interviews to identify recurring themes concerning the permitting process. Further, the interview data provided insights into the duration of the permitting process, capacity building within different jurisdictions, and burdens complicating the application process for installers. This feedback and the analysis enhanced the original permitting model, and the Generalized Permitting Process was developed.

Figure 2 shows the path to approval with the decision points, which, depending on an AHJ’s particular requirements, the complexity of the project, and the details of the application, can lead to additional steps and reviews. The goal is to illustrate the possible variation and complexity within the permitting process and how that variation then grows when compared across jurisdictions. Identical projects at sites with similar conditions but situated in different AHJs will probably move through the workflow or steps differently. For example, one AHJ may require that all commercial EV charging projects go through plan review and a planning commission hearing, while another only requires an electrical permit but has sequential departmental reviews; the workflow illustrates the additional steps and projected time added to the permit approval process for each scenario. Alternatively, two nonidentical EV charging projects within the same AHJ might move through the workflow or steps differently because of the varying complexities of the projects. These decision points also translate back to the time and costs associated with a permit.

The workflow also includes the requirements at each stage, tips for the applicants, and the key stakeholders responsible for each stage. While there is variation across jurisdictions, information about typical requirements helps installers have a starting point for expected requirements while allowing jurisdictions to better understand how their process might be like or different from the generalized process. In the following method section, we describe the permitting document extraction method and the creation of the database.

### Database

We built an end-to-end pipeline to discover EV-charging permitting documents across U.S. jurisdictions, validate and persist the sources, parse unstructured PDFs, and extract structured data fields into a normalized research database aligned to Authority-Having-Jurisdiction (AHJ) levels. Figure 1 shows the database generating process in two stages: **document acquisition** (Figure S1 in the Supplementary Information (SI) shows the parsing algorithm) and **document processing & structured extraction** (Figure S2 in the SI).

### Document acquisition via web scraping

At the document acquisition stage (as illustrated in Figs. 1 and S1), we automated the creation of county-specific search queries concerning EV-charging permits and then submit the queries to the DuckDuckGo API (DDGS)^39^, with the goal of acquiring ranked candidate URLs. The system aggregates and filters the outcomes, subsequently retrieving the associated HTML pages and PDFs asynchronously.

Each downloaded document is subjected to two LLM validation stages: (1) county/state relevance, integrating straightforward name heuristics with an LLM-driven assessment, and (2) EV‑charging content relevance, determined via automated keyword filters and LLM-based review. Documents are retained only after they have successfully passed both checks; basic metadata (source URL, file path, type, county/state, validation status) are recorded, and accepted files are written to the output directory. After the validation process, this dataset is then used as the initial input for the parsing and structured extraction phase (described in the next section).

#### Validation

This process was manually validated by ensuring that a small collection of known EV charging permit process documents could be found by the LLM (ensuring minimal false negatives). The manual validation estimated that we have a false negative rate of approximately 10%. That is, we estimate we missed 10% of relevant documents for AHJs where the document exists (note that this is not 10% of all AHJs). Note, the 10% error pertains to the document retrieval phase. Specifically, when using our specialized Energy Language Model (EV-ELM) to identify relevant documents, 10% of the retrieved items were not EV permitting processes. We acknowledge that it is impossible to ensure perfect retrieval with the LLM, which could bias the results by missing permitting documents in some AHJs, but we attempted to minimize this possibility with our validated retrieval of known documents.

### Document parsing and structured database

Figure 2 illustrates the transition from retrieved permitting documents to a structured database through document parsing. Figure S2 in the SI explains the sequence of the parsing pipeline to retrieve the data tables. First, we transform the downloaded documents into plain text format, employing native PDF extraction techniques and the Optical Character Recognition (OCR) when dealing with scanned documents. We used LLMs to extract EV-charging-related details from various raw text documents, including broad ordinances, city planning documents, and deployment materials. As a result, the extracted content pertaining to EV-charging is preserved as plain-text files, with the document ID serving as the designation for each filename. Every file that is extracted is saved using a distinctive Document ID, formatted as {StateID}_{CountyFIPS}_{Citycode}_{Seq}, which serves the purpose of uniquely associating the document with its respective state, county, or city. The CountyFIPS and Citycode values are extracted from the transportation database^40^. This “*Document ID”* serves as the primary key that links the three tables in the final structured database. 

We developed targeted prompts that are informed by the *Generalized Permitting Process* and the literature review to build three structured tables by employing the decision-tree based LLM queries^14^. The targeted questions were prompted, and the answers were then collected as data dictionaries, which we then converted into structured tabular datasets. The first table in the database provides a comprehensive overview of the entire permitting procedure, encompassing prerequisites, costs, schedules, and stipulations, specifically for electrical, building, and zoning permits and inspections, categorized for commercial and residential projects. The second table encapsulates electrical codes and standards, detailing specifications for equipment, load computations, conductor ratings, and relevant regulations. Finally, the third table shows the ADA standards and building/zoning codes, integrating accessibility, siting, and site-design guidelines. The three tables are available as a dataset at OEDI^35^.

A manual validation exercise on a small subset of documents revealed 95% accuracy for the final output data, which is comparable to previous work^14^. LLMs are known to make mistakes, and we do not expect the outputs to be perfectly accurate. Note that our previous work has found that similar pre-LLM databases developed by purely human efforts have similar accuracy^14^.

### Development of scoring method

The database compiles publicly available checklists, permit documents, and permit applications for EV charging installations. Publishing these materials aligns with several recommended strategies for AHJs to streamline permitting. However, the best practices identified within the literature on permitting EV charging installation lack a scoring method designed to evaluate the clarity and efficiency of the permitting process and the associated application documents published by AHJs. To bridge this gap, we established a set of criteria for evaluating and scoring publicly accessible EVSE permitting documents from diverse AHJs and leveraged the database we assembled with LLMs^35^. Common criteria in existing scoring methodologies encompassed: the creation of distinct permitting checklists for residential versus commercial/industrial installations of Level 2 chargers and DCFCs; an online system for permit submission and approval; acceptance of electronic signatures; transparent approval/review timelines; and the publication of relevant model codes alongside each permitting requirement^36,41,42^.

Building upon earlier efforts in literature, our scoring method evaluates how well the permitting document explains the requirements and describes the due process based on four categories: Process Description, Requirement Explanations, Technology and Tools, and Communication and Support, as shown in Table 1. Each category is weighted to reflect its importance in ensuring the clarity (i.e., the specific details for the application) and the efficiency (i.e., helpfulness of the guidance documents) permitting experience. For each line item within a category, a “*Yes*” or “*No*” is recorded based on whether the feature mentioned appears within the AHJ’s permitting document. A *“Yes”* indicates that the feature is present in the permitting document and contributes positively to the category score. A *“No”* indicates the absence of that feature and does not contribute to the score. Category scores are the sum of the “*Yes*” responses, adjusted by the category weight. Weighted category scores are then summed and scaled to produce a final permitting process score from 0 to 5, where 5 represents an exemplary application and 0 represents an AHJ that does not specifically address EV charging in its permitting application. The Supplementary Information (SI) shows examples of EV charging permitting processes for the City of Southington, Connecticut, and the City of San Diego, California, and how their processes were analyzed and scored to illustrate the range of possible scores^43,44^.

**Table 1:** Scoring method for AHJs’ EVSE permitting application processes.

Permitting application categories and criteria	Category weight scoring
Process description	40%
Permitting application and instructions are specific to EV charging stations	
The permitting process from application submittal through inspections for EV charging installations is described	
Clear step-by-step instructions on how to complete the application are provided	
Checklist for permitting requirements is included	
Permit application outlines the different types of permits that apply to the EV charging project	
Permit application outlines the types of inspections that are applicable to the EV charging project	
Permitting application details the applicant type allowed to submit the application	
Exceptions to any required steps or documentation are outlined	
Review, approval, and inspection timelines are outlined (this review process should take *X* days, you should expect comments back in *X* days, etc.)	
Expedited review eligibility criteria are available and explained	
Any applicable fees are identified and explained	
Requirement explanations	35%
Documents, calculations, plans and other required information are outlined clearly	
The current model codes (IBC, NFPA, NEC, IRC, etc.) followed by the AHJ are referenced	
Site plan and engineering drawing specific requirements are listed in detail (i.e., title page, single line, manufacturer specs, load calcs, etc.)	
The specific code article and section number are referenced with each permitting requirement.*	
Accessibility requirements are outlined	
Example design illustrations for accessible charging parking stall configurations are provided	
Specifies which requirements are needed for different site types (non-residential or residential)	
Application asks submitter to specify the charger type to be installed	
Technology and tools	20%
Online/electronic permit submittal is available	
Online/electronic permit submittal instructions and links are provided in the permit application	
Electronic signatures are accepted	
Communication and support	5%
Contact information for relevant reviewing agencies are included on the application	
	Total score (0–5)

*Signifies that this criterion was not scored using a Boolean approach. Rather, for this criterion within the “Requirements Explanations” section, we assigned scoring based on the bucketed numbers of code references observed. See more details in the Scoring AHJ’s Documents section below.

The **“Process Description” (40% of the score)** scrutinizes the AHJ’s clarity of EV charging permitting procedures for prospective applicants. This category is assessed based on the permitting application’s inclusion of a specific, detailed process for EV charging installation projects and its ease of use. EV charging projects with the scope of permitting applications tailored to them receive high scores, since the inclusion of procedures irrelevant to EV charging installations in generic applications may introduce confusion and unnecessary labor. High-score processes have a clearly defined, step-by-step process checklist with explanatory descriptions for application submission, including inspections, which ensures applicants’ understanding of the initial requirements, thus minimizing errors, revisions, and project delays. Furthermore, estimating timeframes for each phase of the permitting procedure increases the score because they could promote transparency and enable applicants to manage project schedules. Note that the scoring method does not consider whether the AHJ has the capacity or the resources to process the applications faster. However, the expedited review of the applications can reduce the time costs; therefore, the permitting processes with quicker review timelines get higher scores.

The “**Requirement Explanations**” **(35% of the score)** assesses the clarity of the AHJ’s permitting requirements, which results in a high score for the respective permitting process. This involves mentioning relevant construction and electrical regulations, and specifying necessary documents like load computations, site layouts, and equipment details. Clearly stating requirements, along with supporting code examples, helps to prevent errors and the need for revisions, which can otherwise delay the permitting process. An effective and high-scoring permitting process would clarify the specific requirements for various installation types, including Level 2 and DC fast chargers. The requirements should also differentiate between site types, including single-family homes, apartments, and commercial or industrial properties. This granularity ensures that applicants only need to focus on the requirements relevant to their specific project.

The “**Technology and Tools**” **(20% of the score)** assesses the user experience of the permit application process, focusing on expedited submissions, and adds to the score of the permitting process. In this category, AHJs that allow electronic permit submissions (either through email or an online portal) and clearly explain the associated electronic submission process receive higher scores. Electronic permit applications and the use of e-signatures help accelerate the permitting process.

The “**Communication and Support**” **(5% of the score)** scores whether the permit application provides contact information for those involved in its review process. Applicants benefit from opportunities to communicate and resolve application problems or uncertainties promptly.

### Analysis

#### Summary of EV permitting documents in database

**Fig. 3:**
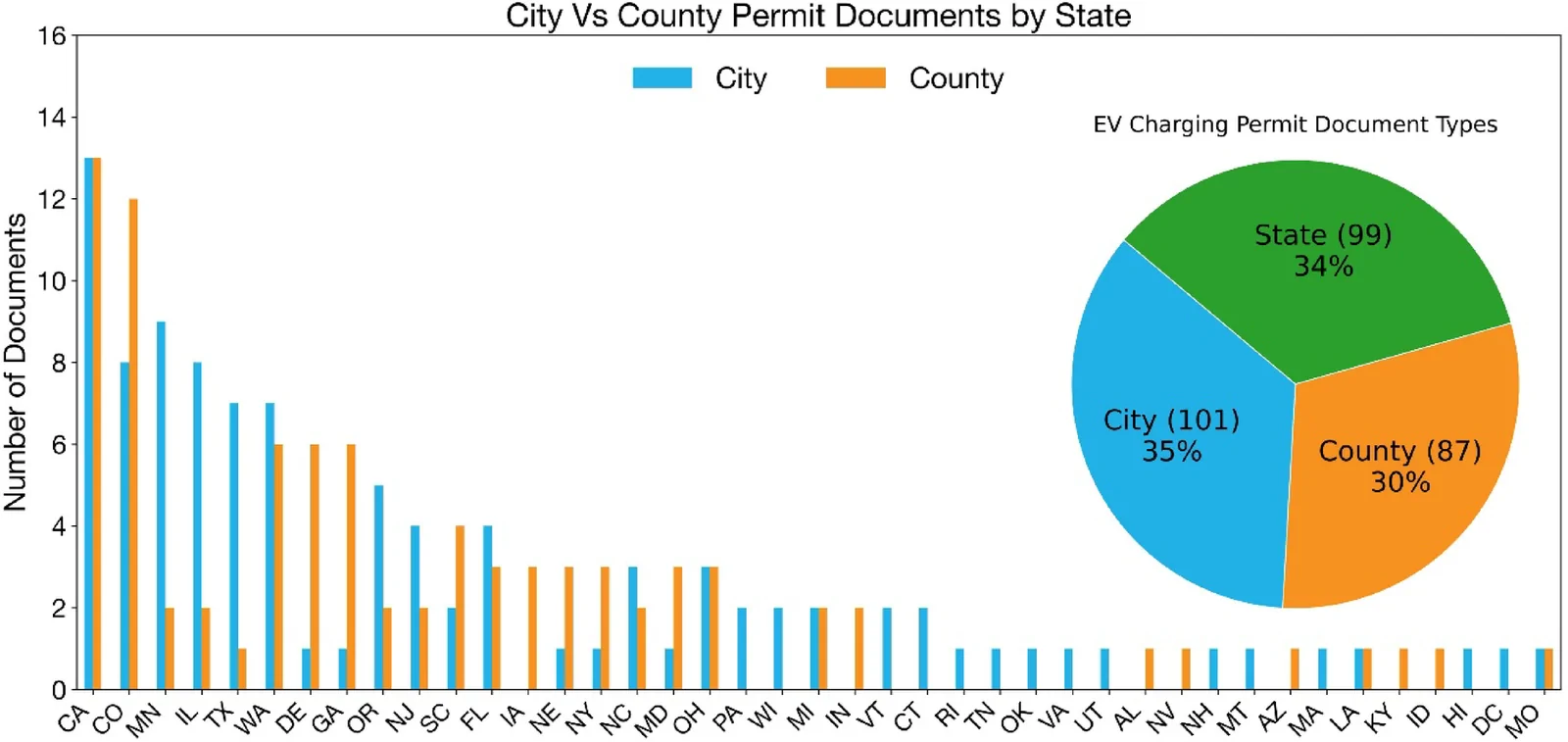
The pie chart shows the distribution of the total number of EV permitting documents in the database. The database contains 287 documents: State (99), County (87), and City (101). The bar chart shows the state-wise distribution of city and county-level EV permitting documents. Note that these charts do not analyze or show whether the AHJ has the capacity or the resources to process the applications faster.

The use of LLMs resulted in the extraction of EV charging permitting documents from across the U.S. As shown in the pie chart in Fig. 3, these documents are distributed across the city, county, and state levels. The data tables comprise 101 city-level documents from 96 cities, 87 county-level documents from 77 counties, and 99 state-level documents from 36 states. Please note that multiple documents (i.e., requirements) exist within the same AHJ (e.g., a state); consequently, there are 99 state-level documents despite only 50 states in the U.S.

In the U.S., some states have prescribed statewide ordinances or codes and some have defined state-level guidance to streamline the permitting processes across the AHJs, and similar steps have been taken by some counties if state-level guidance was not available^36,41^. As shown in Table 2, not all states have state-level prescribed ordinances or codes; conversely, there are some states where the statewide guidance is defined but not the ordinances. However, there are a few states, including California, Delaware, and New Jersey, where both the ordinances and guidance documents are available at the state-level. The pie chart in Fig. 3 shows the distribution of all permitting documents, and the bar chart in Fig. 3 shows the distribution of city and county-level permitting documents across the states in the database. In states without either a guidance document or an ordinance regulating EVSE permitting, the availability of permitting documents at the city and/or county-levels speaks to the local governments’ initiative to be permit-ready regarding EV charging infrastructure.

**Table 2:** List of states showing if the statewide ordinances/codes and guidance documents are available for EV charging permitting processes.

State	Statewide ordinances/codes	Guidance document(s)	State	Statewide ordinances/ codes	Guidance document (s)
Alabama	No	No	Montana	No	No
Alaska	No	No	Nebraska	No	No
Arizona	No	No	Nevada	No	No
Arkansas	No	No	New Hampshire	No	Yes
California	Yes	Yes	New Jersey	Yes	Yes
Colorado	Yes	No	New Mexico	No	Yes
Connecticut	No	No	New York	No	Yes
Delaware	Yes	Yes	North Carolina	No	No
Florida	Yes	No	North Dakota	No	No
Georgia	No	No	Ohio	No	Yes
Hawaii	No	Yes	Oklahoma	No	No
Idaho	No	No	Oregon	No	Yes
Illinois	No	No	Pennsylvania	No	No
Indiana	No	No	Rhode Island	No	No
Iowa	No	No	South Carolina	No	No
Kansas	No	No	South Dakota	No	No
Kentucky	Yes	No	Tennessee	No	Yes
Louisiana	No	No	Texas	No	No
Maine	No	No	Utah	No	Yes
Maryland	No	No	Vermont	Yes	No
Massachusetts	No	Yes	Virgina	No	No
Michigan	No	Yes	Washington	No	Yes
Minnesota	No	No	West Virginia	No	No
Mississippi	No	No	Wisconsin	No	No
Missouri	No	No	Wyoming	No	No

Figure 3, in bar chart, presents the quantity of EV-permitting documents acquired at both the city (blue) and county (orange) levels for each state. California contributes the largest volume at both county and city levels, consistent with its governor’s effort to streamline permitting^36,41^. County-level documents are highly prevalent in Colorado, with a strong number of city-level documents as well, consistent with Colorado’s effort to streamline land-use codes and associated permitting for the EV charging stations^45^. Minnesota, Illinois, and Texas, states with no available guidance or ordinance documents, follow with high numbers of city-level documents. The presence of state-level documents is not necessarily indicative of the presence of local-level permitting documents. New Jersey, for example, is a state that has both state-level ordinances and local guidance documents and falls lower on the document count. This, perhaps, is an indication that the AHJs within the state may not feel the need to create their own ordinances and instead can simply adopt the state guidance to make the permitting processes uniform. However, in the case of some states, the lack of smaller geographies (fewer jurisdictions within the state) besides the time and effort required to change local ordinances and develop permitting documents to align with statewide goals. The extended right tail, encompassing numerous states with few documents, underscores inconsistencies in the public availability and searchability of these documents.

**Fig. 4:**
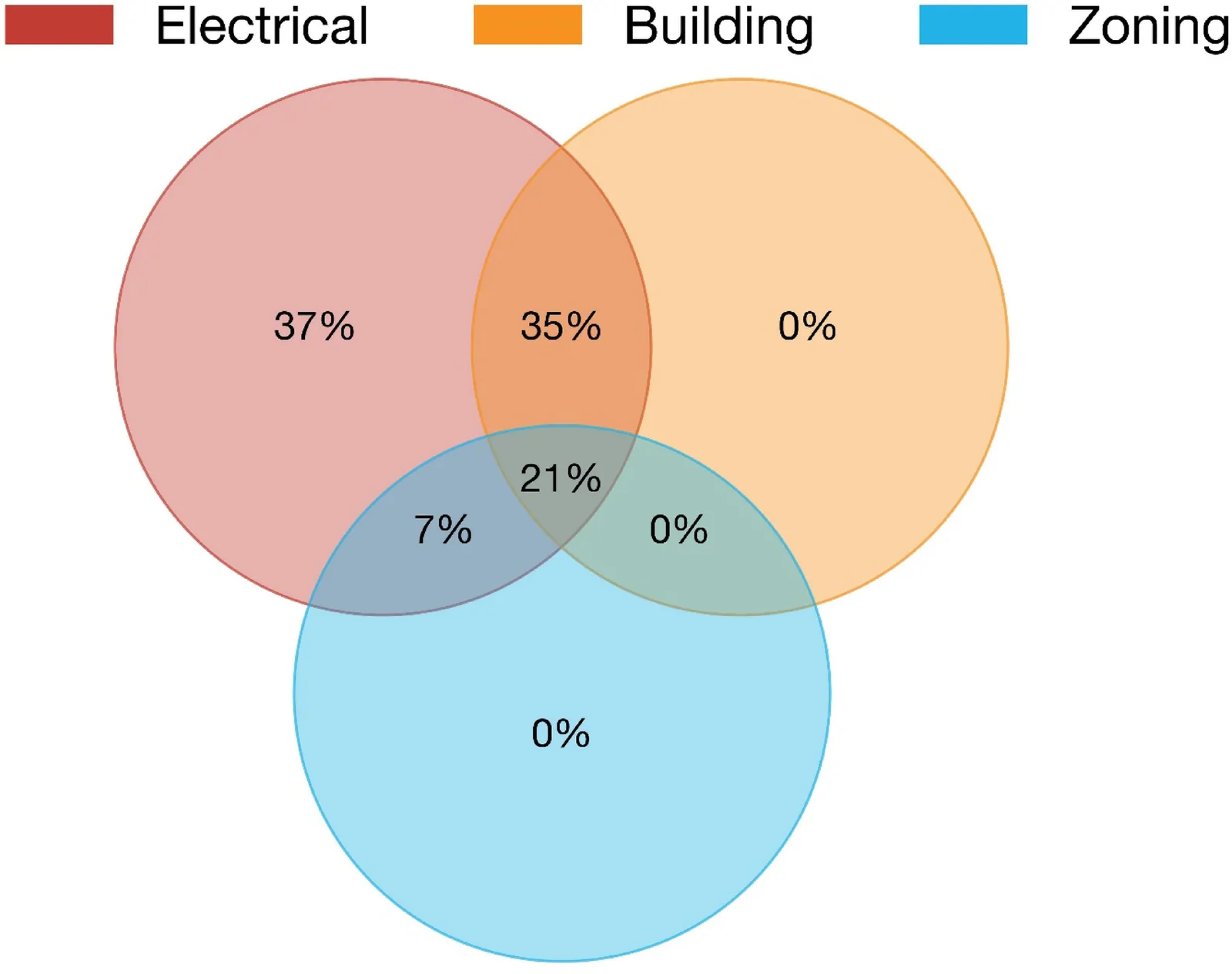
Venn Diagram of the number of EV permitting documents referencing the three main permit (i.e., Electrical, Building, and Zoning) requirements. 21% of the documents reference all three permit requirements. Out of the three, “Electrical” is the most commonly required permit. Note that this diagram does not show whether the AHJ has the capacity or the resources to prepare the permitting documents that would precisely define the permitting requirements.

The Venn diagram (Fig. 4) summarizes which permit types AHJs mention in the EV charging permitting documents. An electrical permit is referenced by almost all AHJs, with 37% of them mentioning only electrical permits; however, building and zoning are required in conjunction with electrical permits. 35% of the EV permitting documents mention both electrical and building permits, whereas 7% mentioning electrical and zoning permits and 21% require all three permit types.

Considering the responses, all the AHJs mention electrical permitting, while roughly 56% bring up building permits (which are always with electrical), and approximately 28% reference zoning (which never exists independently for EV charging stations). Practically, this shows that the electrical permit is the universal and critical permit requirement for EV charging installations, while a combination of building and zoning permits, and subsequent reviews, are required in a subset of jurisdictions.

The installation of EV chargers requires adherence to two distinct types of local oversight: adherence to locally adopted model codes (i.e., building and electrical codes), and adherence to local regulations (i.e. zoning, land use). Consistent with literature findings, the permit documentation clearly defines the straightforward processes for complying with building and electrical codes. This simplicity arises because these codes are derived from standardized model codes that come with established review frameworks. The primary challenge lies in local zoning and development regulations, which according to the literature are more closely tied to the lack of clarity on EV charging regulations^17,24^ and therefore influential in defining the process for EV charging permitting (as articulated in the flowchart). These regulations often lack explicit provisions for EV charging, creating ambiguity and leaving their application during the permitting process to the interpretation of local officials. This leads to significant inconsistencies across jurisdictions^24^. Ambiguity in the permitting process regarding EV charging installations may explain the inconsistent appearance of zoning regulations in related documents (Fig. 4), despite these regulations influencing the design and placement of such installations.

The literature is clear that while a zoning permit may not always be required, zoning or land use reviews may still be a part of the electrical or building permit process. One example of a prevalent challenge in zoning codes is failing to address the relationship between EV charging and parking requirements, which can significantly prolong the permitting process^24^. Zoning codes often set minimum parking requirements based on the type of land use and how that property is zoned^17,24^. The installation of an EV charger in an existing parking space has the potential to result in non-compliance with the minimum parking requirements of the establishment, which could spark a need for a parking variance or other regulatory processes in order to proceed with the installation as designed^15,17,24,29,33,36^. The permitting documents analyzed through this research effort point to a discrepancy between local regulations and what regulations are explicit in the permitting documents.

This discrepancy is reinforced by actions planning entities are taking to better define how local regulations should adapt to provide the appropriate level of land use, zoning, or development regulations for EV charging and then to clarify what those regulations are and how they apply to various types of EV charging installations. For example, the state of Colorado identified this challenge and, as a result, developed a model land use code specifically for EV charging that is tied to specific requirements for adoption by certain Colorado jurisdictions. The Colorado Energy Office outlined a recommended permitting process for EV charging which includes a land use review as the foundational first step for certain EV charging projects (in this case, only projects where EV charging is the primary land use), preceding applications for building or electrical permits. This newly established practice was created specifically to resolve the procedural and documentation gaps that previously existed for EV charging land use permitting^46–48^. Consequently, while the LLM successfully retrieved official EV permitting documents, it likely failed to capture the associated zoning and land use requirements. This occurs because those regulations are often omitted from the documents themselves, despite being mandatory in practice.

### Keywords analysis

**Fig. 5:**
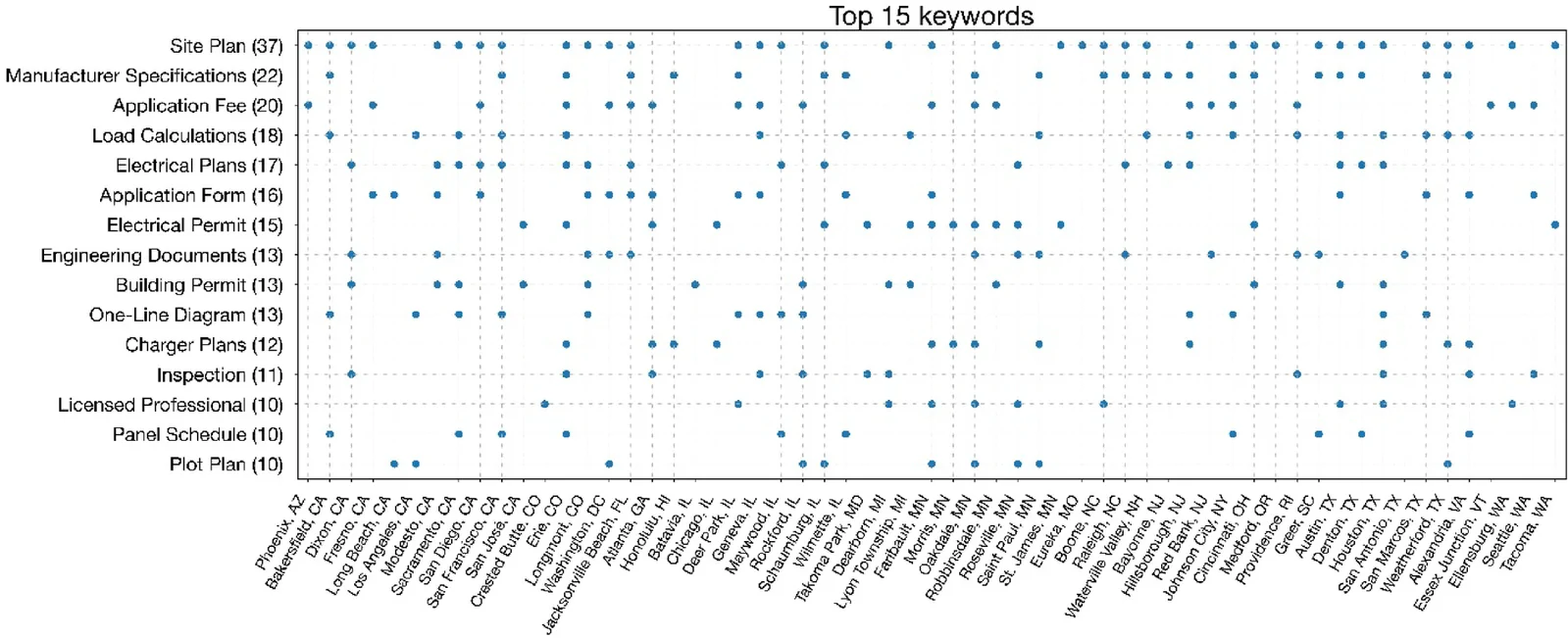
Most common keywords in a typical permitting document and the cities with their frequency. The y-axis labels of the figure represent the count of unique cities where the keyword was present, and the x-axis displays the cities within each state. The number in the parenthesis shows the frequency of the keywords. Further, the ‘dot’ in the graph shows that if the particular keyword is mentioned in the respective city’s EV charging permitting process. “Site Plan” refers to a detailed drawing of a property for the EV charging installations. “Manufacturer Specifications” refers to the technical specifications of the equipment by the EV charging manufacturer. “Application Fee” refers to the fees required for the application. “Load Calculations” refers to the load calculations showing if the electrical infrastructure supports the additional load of EV charging stations. “Electrical Plan” is a detailed technical drawing. It shows a building’s electrical system layout and specifications with symbols, lines, and annotations. “Engineering Documents” refers to technical records detailing a project’s design and specifications. “One-line diagram” refers to the simplified schematic of the power and electrical system. “Plot Plan” refers to a map or visual representation of a property, displaying the positions of significant components including structures, property edges, access roads, and landscaping. “Application Form” is a standardized document used to officially request a permit. “Licensed Professional” refers to a professional with the authority and required training.

We, further, analyzed the top 15 keywords most frequently appearing in the answers derived from city-level EV charging permitting applications. We used the Yet Another Keyword Extractor package^49^ to extract the keywords from the database and used Natural Language Toolkit^50^ to remove the extra words and streamline the keywords. The keywords depict what requirements are defined within the EV charging permitting documents, and they are displayed in Fig. 5. As shown in Fig. 4, the electrical and building permits appear more often compared to the zoning permit. However, the most common keyword is “site plan” in the EV permit applications, highlighting the work required from the installers to prepare their site plans. Similarly, “Engineering Documents” and “Plot Plan” are related to the “site plan” documents. The “site plan” is, importantly, evaluated during the inspection of the construction, and “inspection” is also another entry in these top 15 keywords. “Manufacturer specifications of charging stations”, “load calculations”, “panel schedule” and “electrical plans” and “one-line diagrams” are related to the electrical systems of the EV charging installations.

The past studies have also shown that the absence of unambiguous and uniform direction concerning ADA mandates for the installation of EV charging stations is a contributing factor in the permitting procedures^24^. However, the Fig. 5, which shows the keywords, does not show any specific details related to ADA requirements. The ADA sets nationwide accessibility rules, including both public and private parking. Because of the vagueness of this situation, installers are unsure of local ADA rules, leading to design changes and permit approvals^24^.

### Insights into permitting strategies using LLM

The current body of work on EV charging permits often prescribes best practices or policy recommendations focused on expediting the permitting process. Various studies have introduced scoring criteria and checklists intended to help AHJs optimize their EV charging permitting processes^36,41,42^, and often the AHJs’ performance will be gauged by how well they implement the recommended actions for the EV charging permitting process. The suggested actions might encompass the development of checklists for EVSE permitting, accelerating the review and inspection processes for EVSE projects, and modifying or enacting zoning ordinances that support EVSE installation^36,41,42^. Table 3 summarizes the best practices or strategies to mitigate specific permitting challenges and the benefits of the prescribed strategies. We, further, analyzed the LLM-generated database to find the evidence of the measures that have been implemented by the AHJs.

**Table 3:** Documented Best Practices for EVSE Permitting and Insights from the LLM EVSE Database.

Strategy	Challenge addressed	Benefit	Source	Evidence in the LLM-generated OEDI database
Online permit application with electronic signature	In-person permit submissions restricted to certain hours of the day/week	Easier submission for installers. Can ensure that all components of application are included before submission	^15,17,24,29,34,36^	25% of documents allow for an online application
Post checklist and best practice guidance on AHJ website- specific to EV charging permit process	EV charging installation is new and often not addressed explicitly under electrical or building permits, leading to ambiguity about the requirements and possible timeline	Avoids ambiguity for staff reviewing the permit application and for those submitting the application. Ensures more complete applications that meet requirements for approval	^15,17,24,29,34,36^	Gathered and documented through the LLM database, when they exist
Use specific EV charging permit or permit process	EV charging installation does not fit neatly into the standard electrical or building permits, meaning if submitted through these permits the AHJ may not have all the information they need	Enables specificity on design, site plan, and other local guidelines. Minimizes need for resubmission by designing clear ask for required information upfront	^15,24,36^	Many cities and counties do not have an EV charging specific process, regardless of the presence of state-level guidelines or ordinances
Amend zoning code to define which level of charger can be approved administratively in each zone. When possible, permit as accessory use in all zones	Many zoning codes do not address EV charging specifically, leading to ambiguity and in some cases classification as a fueling station which is highly restrictive and may require public hearing	Administrative review and clear codes avoid long review process and the potential for a public hearing or planning commission hearing. Provides clarity to reviewers and applicant	^17,24,33–36^	28% of documents mention a zoning permit requirement but are less detailed in which zones the EV charging installations are allowed
Concurrent department reviews of permit	Extensive and sequential reviews may unnecessarily extend the timeline for permit review	Eliminates sequential reviews and reduces overall time for review process	^17,24,29,34,36^	61% of documents indicate that plan review (involving departmental reviews) is required for EV charging installations. Does not indicate if reviews are sequential or concurrent
Communicate and standardize ADA requirements	ADA requirements for EV charging are not standardized nationwide	Ensures clarity for installers and more accessible infrastructure. Plan review will be more efficient if AHJ has standardized requirements	^24^	Less than 20% of the documents mention ADA or accessibility requirements specifically. Dataset details specifics for those documents that do mention accessibility criteria
Establish policy enabling EV charging parking spots to count towards minimum parking requirements	An EV charger may “take” a parking space, causing an establishment to no longer meet minimum parking requirements	Clearly defining how EV charging spots relate to minimum parking standards avoids ambiguity in the design and permit process. Policy attributing (up to a certain percentage) of EV charging spots to qualify as one or more parking spaces facilitates charging installation	^15,17,24,34^	EV policy on parking spots as it relates to parking minimums not explicit in permitting documents. 14% of documents mention other codes or requirements related to parking
Address EV infrastructure in planning documents	As a relatively new land use, there is ambiguity in how to understand its impact on surrounding areas, and in which zones it is an appropriate land use	Provides guidance/ awareness for planning staff reviews. Eliminates ambiguity in how to interpret AHJ vision or goals	^17,24,36^	Developing an EV charging permitting document, like the ones documented in the LLM, is a tangible strategy by which AHJs might implement EV charging infrastructure goals outlined in planning documents

“Strategy” recommended by different “Sources”, that would address the “Challenge” in the permitting processes, and the “Benefits” of the respective strategy. Lastly, the column “Evidence in the LLM-generated OEDI database” shows if these strategies are implemented by the AHJs in their permitting processes. Note that the strategies are not listed in any specific order of priority or preference.

### Scoring the AHJs’ documents

We applied the scoring method to the EV charging permit documents database, in which each document was assessed according to whether it contained certain criteria or pieces of information. When possible, we looked at Boolean answers and gave them points depending on whether the response was accurate or favored. Generally, a positive score for a document in the EV charging permitting database was linked to a non-empty or affirmative answer. For the “Requirement Explanations” section, a scoring system was used to evaluate whether the permit application properly identified the code article and section number relevant to each requirement. Since several database questions related to this, the answers were categorized, and points were given based on the range each document fit. Documents that did not reference any specific codes received zero points; those referencing one to three code requirements received one-third of the available points; documents referencing three to five received two-thirds; and documents referencing over five distinct code requirements received full points. Note that this process uses the LLM to identify and record features of the permitting document in a structured format, which we then use for subsequent quantitative scoring. We do not use the LLM to subjectively score the quality of documents, as this would be much more prone to opaque and error-prone LLM decision-making.

## Results and discussions

**Fig. 6:**
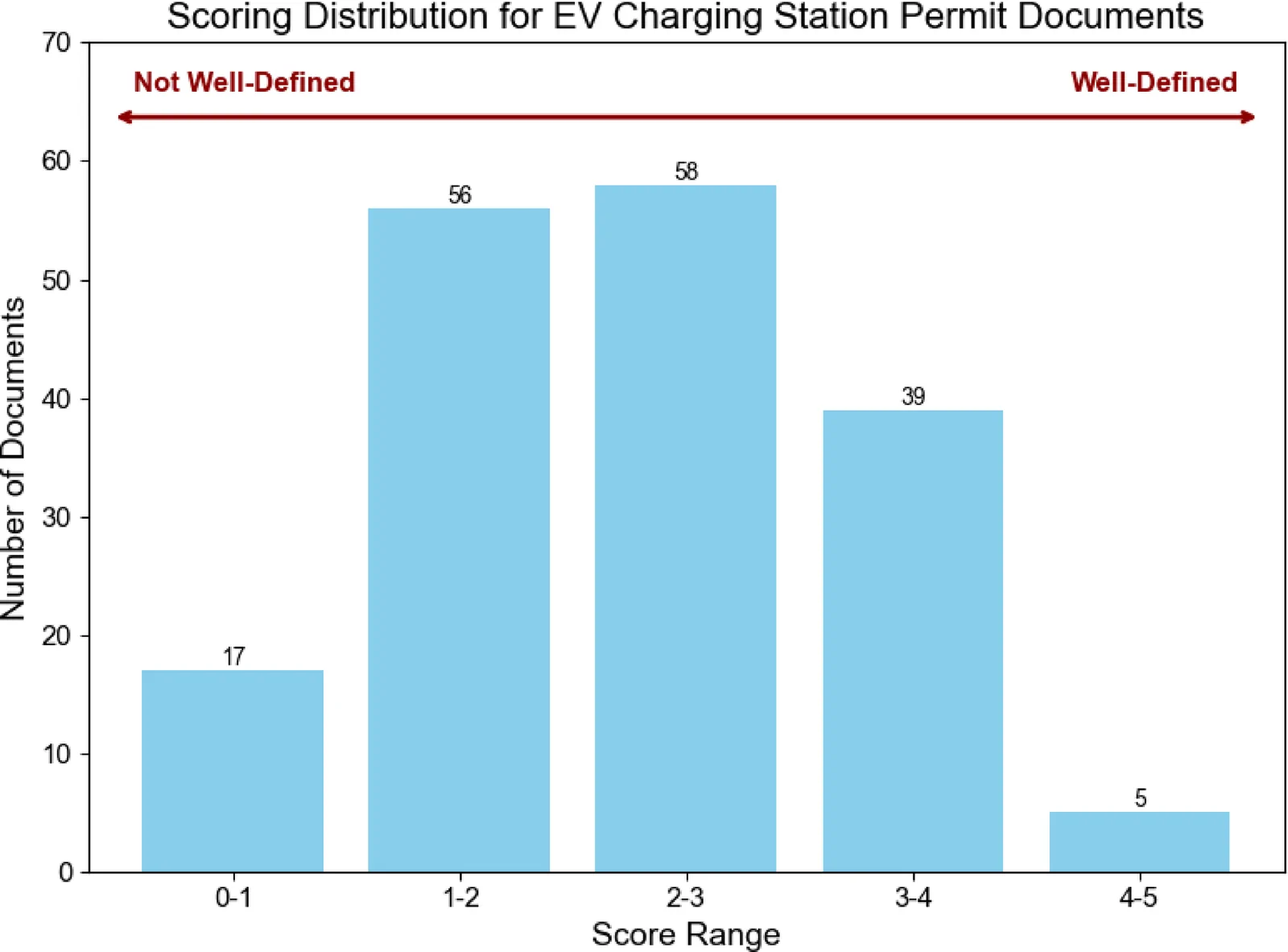
Histogram of the Scoring Method Results. The documents scored are permitting documents representative of different locations (on most accounts, each jurisdiction has one EV Charging permitting document represented in the LLM; however, some locations have multiple relevant documents). Consequently, the count of documents within each score range indicates how scores are distributed among locations with EV charging permitting documents. While the scoring method highlights the completeness and definition of the process within the documentation, it does not accurately represent the real-world permitting experience in the given locale.

Figure 6 shows the distribution of scores of the EV charging permitting processes across the evaluated dataset. Note, we did not use LLMs to score the documents, but we used the structured database generated by the LLMs for scoring analysis. Approximately 42% of the documents (n = 73) scored below 2, while only a small number (n = 5) achieved scores in the 4–5 range. This distribution suggests that the scoring framework is relatively stringent, with higher scores showing an exceptional level of completeness and clarity in permitting documentation. Across all 175 local permitting documents analyzed (i.e. excluding documents that were state-level guidance and those not focused on EV charging station permitting), the average score was 1.8. Overall, these results indicate that many jurisdictions still provide limited or inconsistently detailed EV charging permitting information, underscoring the need for more comprehensive documentation to support clear and efficient permitting processes.

Table 4 summarizes the ten lowest- and ten highest-scoring sections, the frequency with which each section received points (out of the 175 documents scored) and the maximum possible score for that section. The lowest-scoring sections, which indicate frequent omissions in permit documentation, included topics such as inspection costs, zoning reviews, and building permit requirements, as well as other inspection-related details. In our research, we found that these topics are less likely to be included in a permitting document. In contrast, the highest-scoring sections addressed elements such as specifying the use of the EV charging installation, identifying a permit contact, including a process checklist, and providing information related to service upgrades. We found these topics are more likely to be included in a permitting document through this scoring process. The table highlights trends, with the goal of identifying opportunities for improved process descriptions within permitting documents.

**Table 4:** 10 Lowest- and Highest-scoring Sections.

Section	Frequency awarded	Max section score
Least frequently awarded points		
Project stage or trigger after which a plan review is required for commercial EV charging installations (Table 1 row 51)	0/175	0.02
Notes on expected timelines or duration for inspections in commercial EV charging installations (Table 1 row 89)	4/175	0.03
Notes on zoning-review fees or cost components for commercial EV charging installations (Table 1 row 70)	5/175	0.03
Notes on inspection fees or cost components for commercial EV charging installations (Table 1 row 88)	6/175	0.03
Indicator of whether a mechanical inspection is required for commercial EV charging installations (Table 1 row 82)	6/175	0.05
Conditions under which inspections are not needed for commercial EV charging installations (Table 1 row 87)	7/175	0.03
Notes on expected timelines for the zoning-review process for commercial EV charging installations (Table 1 row 71)	7/175	0.03
Boolean indicator that the content addresses residential use cases (e.g., single-family homes, home garages) (Table 1 row 14)	8/175	0.07
Notes on building-permit fees or cost components for commercial EV charging installations (Table 1 row 41)	10/175	0.03
Conditions under which a zoning review is not needed for commercial EV charging installations (Table 1 row 69)	11/175	0.03
Most frequently awarded points		
Boolean indicator that the text covers general, non-EV specific permitting regulations or laws (Table 1 row 7)	175/175	0.18
Indicator of whether a plan review is required for commercial EV charging installations (Table 1 row 50)	132/175	0.06
Boolean indicator of whether the document includes a checklist for the commercial EV permitting process. (Table 1 row 117)	130/175	0.18
Boolean indicator that the content addresses commercial use cases (e.g., public, retail, workplace, multifamily, federal, shopping centers) (Table 1 row 13)	129/175	0.07
Name or entity serving as the primary contact for the commercial EV permitting process. (Table 1 row 112)	123/175	0.01
Indicator of whether a building permit is required for commercial EV charging installations (Table 1 row 37)	121/175	0.06
Project stage or trigger after which an electrical permit is required for commercial EV charging installations (Table 1 row 25)	119/175	0.02
High-level sequence of steps in the commercial EV permitting process (concise, 3–4 key actions) (Table 1 row 115)	117/175	0.18
Indicator of whether a zoning review is required for commercial EV charging installations (Table 1 row 64)	116/175	0.06
Requirements and reviews for service upgrades/new service tied to commercial EVSE electrical work (Table 1 row 27)	101/175	0.03

How many documents were awarded a point for the specific scoring category defined by the research team. Low frequency sections are indicative of information that the research team found is less likely to be found within a permitting document. High frequency sections are indicative of information that the research team found is more likely available in permitting documents. (Table and Row numbers within parentheses refer to the Open Energy Data Initiative (OEDI) database^35^).

### Implications of the scoring method

The scoring method developed here provides the first systematic, well-defined approach for evaluating EV charging permitting documentation quality. Unlike existing scoring criteria that are vague or process-focused, our method quantifies the clarity of AHJs’ permitting application requirements, rather than evaluating the quality of the underlying permitting process itself. This distinction is crucial. Clear and effective documentation can significantly reduce ambiguity—a factor that stakeholders consistently identified as costly in terms of both time and resources — and improve applicant responsiveness to the AHJ’s requirements.

The scoring method has immediate practical uses, including as a diagnostic tool for AHJs to identify where their constituents would benefit from targeted improvements to their permitting documentation. For jurisdictions without existing EV charging-specific permitting processes, the scoring criteria can serve as a development guide, ensuring that new documentation addresses the most critical elements for successful permit applications. As one stakeholder noted, *“Simplicity is still okay, but ambiguity can be costly,”* emphasizing that even straightforward processes benefit from clear documentation.

### Database insights and LLM performance

The process of creating a database using LLMs revealed important information about EV charging permitting documents and the abilities of LLMs when used for policy research. The LLM’s proficiency in handling varied document types and formats is demonstrated by the successful extraction and organization of 287 documents from three different jurisdictional levels (city, county, and state). Based on our review of the literature, this study is the broadest assessment of EV charging permitting applications and guidance documents in the U.S. and was made possible using LLMs.

However, our examination also showed some key constraints in the capabilities of LLMs. As noted in the validation, the performance of the LLM in finding and extracting information from the relevant documents was validated manually (i.e., by a human) for a small subset of the total documents. We estimate that the final database should be approximately 95% accurate and AHJs with relevant documents were missed at approximately a 10% false negative rate. This is comparable to previous results that showed 85–90% accuracy in LLM-driven data retrieval^14^. Specifically, the 10% error rate is related to how documents are retrieved. When our specialized Energy Language Model (EV-ELM) was employed to find related documents, it was found that 10% of the results were not about EV permitting processes. The data extraction from correctly recognized EV permitting documents showed a 5% parsing error rate. This shows that in 5% of confirmed relevant documents, the model encountered issues with data parsing or extraction, resulting in inaccuracies. We expect such results should be sufficiently accurate for downstream research but want to express that such a database can likely never be perfectly accurate. Our previous work has found that similar pre-LLM databases developed through purely human efforts have similar accuracy^14^. The LLM proved most effective when permitting documents contained explicit, well-structured information. Documents with clear headings, standardized terminology, and logical organization yielded more accurate extractions. Conversely, the LLM struggled with ambiguous language, implicit requirements, and documents where EV charging permitting was embedded within broader regulatory frameworks. This performance variation aligns with the scoring results, where documents with higher clarity scores typically provided more reliable data extraction.

The database also identifies limitations in the availability of documentation. While electrical permits were mentioned in 99% of documents, critical elements like inspection timelines (mentioned in only 4 documents) and zoning permit costs (5 documents) were frequently absent. Based on this trend, many AHJs prioritize fundamental needs, overlooking the procedural precision that could simplify the permit process.

Note that although we believe the database will be sufficiently accurate for subsequent regional EV charging research, we do not guarantee perfect accuracy of the database. In fact, on the contrary, we can guarantee that the database is *not perfectly accurate*, as LLMs are known to make mistakes and will have done so here. Interested readers should not rely on singular values from the database, especially not for decision-making on the permitting activities of real-world charging installations. We have included the database flowchart in the OEDI database^35^ for the readers of this work to navigate the database. Developers and installers, however, should always consult local permitting laws directly with the help of appropriate legal counsel.

### Strategies to improve AHJs’ permitting processes

Several strategies emerge for AHJs seeking to improve their permitting documentation.

Basic:

- Develop EVSE-specific checklists and application processes rather than relying on generic electrical or building permit procedures
- Provide contact details for all departments involved in the review process
- Present instructions, in a step-by-step format, for application completion and submission
- Specify the timelines for each stage of the review process

Intermediate:

- Reference specific code articles (e.g., NEC, IBC, local zoning codes, environmental regulations) and sections for each requirement
- Distinguish requirements for different installation types (residential vs. commercial, Level 2 vs. DC fast charging)
- Include accessibility requirements with illustrative examples to aid interpretation
- Implement electronic submission capabilities with e-signatures and clear instructions describing the submission process

Advanced:

- Provide detailed site plan and engineering drawing requirements
- Include fee structures and payment processes
- Outline expedited review criteria and exceptions to any required steps
- Address zoning considerations and parking requirement interactions

An AHJ may reference example documentation from other AHJs that have already implemented these strategies by referring to the OEDI dataset’s Table 4^35^ that includes the scores as we estimated for this study, and use the *“document_id”* to locate the detailed information in the structured database.

### Limitations

Several limitations should be acknowledged in interpreting these results. First, the scoring method evaluates documentation quality rather than the actual permitting process efficiency or timeline outcomes. While we hypothesize that clear documentation likely correlates with more efficient processes, this relationship requires empirical validation through future research that correlates scoring results with actual permitting timelines and costs.

Second, the LLM extraction process, while comprehensive, may have missed nuances in local permitting requirements or failed to capture informal processes that exist but are not well-documented. As previously discussed, LLMs are known to make mistakes in the interpretation of complex procedural documents, and therefore no one should rely solely on the permitting process database to inform their own real-world installations. The reliance on publicly available documents also means that some jurisdictions with effective processes may be underrepresented if their documentation is not easily discoverable online.

Third, this study is solely focused on permitting documentation instead of examining the wider EV charging installation deployment ecosystem, which organizations like the Interstate Renewable Energy Council address comprehensively through programs like ChargeSmart^42^. Future research could expand this framework to evaluate other aspects of the deployment process.

### Future work

This study establishes the foundation for several promising research directions. For example, if we compare AHJ scores to actual permitting times and costs, we can confirm that the quality of documentation affects how efficient the process is. This kind of analysis could offer real-world data showing how better documentation standards boost returns for cities and their constituents. By validating the relationship between permit scores and installation times and costs, future charging infrastructure needs analyses could quantify the benefit of efforts to streamline permitting to serve demands from vehicle fleets.

This work’s AI-driven approach could also be applied to examine the permitting processes for new technologies, like electricity storage or grid upgrades required for large load management, where local approval issues could similarly curtail installation of new energy infrastructure. In addition, this framework facilitates temporal analysis, enabling the tracking of improvements in permitting documentation quality over time, with a focus on how state-level policy changes influence these improvements. Ultimately, the LLM-based extraction method can be improved and used to study how different areas adjust their permit procedures in reaction to technological advancements, offering a better understanding of how regulations evolve and spread.

## Conclusion

The permitting processes governing the addition of electric distribution grid-connected loads vary across authorities having jurisdiction (AHJ) in the U.S., and the Generalized Permitting Process synthesizes these variations into a simplified process flow. By using Large Language Models (LLMs), we constructed a structured database to explicitly present the variations in permitting processes and their prerequisites. The novel scoring method introduced in this study evaluates the clarity and comprehensiveness of an AHJ’s processes and requirements within their permitting applications.

This study uniquely offers a systematic evaluation mechanism for assessing local alignment with permitting streamlining initiatives. States could potentially use these scores to pinpoint regions that would benefit from more technical or human resources or to assess the pace of installations. For instance, some states have both ordinances and guidance documents, while cities and counties within those states exhibit comparatively few local permitting documents. While the study found deficiencies in the quality and completeness of local electric vehicle supply equipment permitting documentation, it can also inform targeted improvements that improve the effectiveness of efforts. The framework can guide the development of model ordinances and guidance documents that address frequently overlooked components, leveraging practices from high-scoring permitting documents. Thus, the database and scoring method offer unparalleled resources for developers, AHJ staff, researchers, policymakers, and other practitioners to accelerate electric infrastructure permitting necessary to meet growing energy demands.

## Supplementary Information


Supplementary Information.


## Data Availability

Renganathan, Umapriya, Reid Olson, Ranjit Desai, Grant Buster, and Noah Frey. LLMs for EV Infrastructure Permitting. National Laboratory of the Rockies (NLR), September, 24, 2025. Distributed by Open Energy Data Initiative (OEDI). [https://data.openei.org/submissions/8540] Github repo: [https://github.com/NREL/EV-ELM].
