# Supplementary material for: An AI-driven framework for evaluating local and state authorities’ permitting processes
Source: Sci Rep. 2026 Jun 16;16:27454. doi: 10.1038/s41598-026-53770-3 (PMC13534710; doi:10.1038/s41598-026-53770-3)
Supplement: Supplementary file 1 [file 41598_2026_53770_MOESM1_ESM.docx]

Supplemental Information

An AI-Driven Framework for Evaluating Local and State Authorities’ Permitting Processes: Installing Charging Infrastructure

Ranjit R. Desai^1,*^, Umapriya Renganathan^1^, Reid Olson^2^, Erin Andrews-Sharer^1^, Noah Frey^2^, Alexandria Danner^1^, Grant Buster^2^, Jack Titus^3^, and Noel Crisostomo^3^

^1^Center for Integrated Mobility Sciences, National Laboratory of the Rockies, 15013 Denver West Parkway, Golden, CO, U.S.A

*Correspondence: [Ranjit.Desai@nlr.gov](mailto:Ranjit.Desai@nlr.gov)

*Corresponding author

^2^Strategic Energy Analysis Center, National Laboratory of the Rockies, 15013 Denver West Parkway, Golden, CO, U.S.A

^3^ Office of Policy, United States Department of Energy, 1000 Independence Avenue, Washington, DC, U.S.A.

# Methods

Figure S1. Flowchart of web scraping pipeline. In the document acquisition stage (Figure 1 in the manuscript) we build county-specific EV‑charging permitting search queries and submit them to the DuckDuckGo API (DDGS) to obtain ranked candidate URLs. We fetch the top results and apply simple filters (for example, removing obvious non-government domains and known aggregators) before downloading. We then asynchronously download the linked HTML pages and PDFs with sensible timeouts and retry behavior. Downloaded content is first written to a local temporary cache and subsequently normalized (text extraction and cleaning) to support downstream parsing. A lightweight preprocessing pass extracts visible text, normalizes whitespace, and computes document-level statistics (e.g., word/link counts and extracted titles). The normalized text is split into pages/chunks and used in later checks. Each document passes through two validation layers. The first layer addresses county/state relevance and combines deterministic heuristics (ngram name checks and domain heuristics) with structured LLM validators that return JSON-formatted assessments on geographic relevance. The second layer addresses EV‑charging content relevance and applies automated keyword/acronym/phrase filters followed by an LLM-based classifier that evaluates whether the document substantively addresses permitting, standards, or ordinance language relevant to EV charging. Only documents passing both checks are retained. Validation outcomes and anomalies are emitted to the application logging system (including per-location logs). Heuristic scores are recorded in logs as debug messages, and parsed LLM outputs drive decision logic. Basic metadata (source URL, file path, type, county/state, validation status and timestamps) is recorded alongside each document, and accepted files are moved into the output directory as the curated input set for parsing and structured extraction. This implementation is a modified variant of the “ordinance_gpt” example in the open-source ELM repository (<https://github.com/NREL/elm/tree/main/examples/ordinance_gpt)> [1] and follows the approach described in Buster et al. (2024) [2].

Figure S2. Flowchart of the Permitting document parsing pipeline. This figure summarizes the workflow for converting raw jurisdictional permit documents (PDF/TXT) into structured EV charging permitting data and populating the database. Documents are ingested from state/county/city folders, with OCR automatically applied when PDFs are scanned. The extracted text is then normalized and cleaned (e.g., whitespace reduction, deduplication, and removal of irrelevant content). Using Azure OpenAI GPT-4, only text relevant to EV charging installation and permitting is extracted and saved as a standardized parsed TXT file. Next, Azure OpenAI GPT-4 is run through decision-tree–based prompts to extract permit requirements as structured responses. These outputs are captured and written into curated tables aligned with the database fields. For clarity and reuse, results are organized into separate outputs for overall permit/workflow fields, electrical requirements, and other code/checklist categories (e.g., ADA, zoning, and structural).

The Figure S2 follows the steps below :

- **Input:** The process starts with a document, which can be in either text (.txt) or PDF format.
- **Text Extraction:** The text is extracted from the PDF document.
- **Data Cleaning:** The extracted text is cleaned by removing unnecessary spaces and content.
- **Permit Data Extraction:** Specific data related to EV (Electric Vehicle) charging permits is extracted.
- **Parsed Document Storage:** The extracted permit data is stored as a text file, referred to as “Parsed docs”.
- **LLM Application:** Large Language Model (LLM) calls are applied using decision trees.
- **Output and Storage:** The output from the LLM calls is extracted and stored into different code tables in the OEDI database [3]:
  - Overall Permit table (Table 1)
  - Electrical code table (Table 2)
  - Other code table (Table 3)

## Scoring Method Application

This section presents an analysis and scoring of two example Authorities Having Jurisdictions (AHJs) to demonstrate the variability in permit application scores. Included in the scoring are the AHJs: the City of Southington, Connecticut, and San Diego, California [4,5]. The scoring system spans from 0 to 5; a score of 5 signifies a thorough permit application that contains all essential data concerning the EV charging permit procedure, while a score of 0 denotes a complete lack of information regarding EV charging permits. The final score is determined by five evaluation categories, each assigned a specific weight: Process Description (40%), Requirement Explanations (35%), Technology and Tools (20%), and Communication and Support (5%). Each category is evaluated based on predetermined criteria, employing a *“Yes”* or *“No”* response system, with a *“Yes”* answer yielding one point towards the category's total score and a *“No”* answer yielding zero. The total scores for each category are weighted and then adjusted so that a perfect score equals 5. The score of 1.6 was assigned to Southington because the permitting document lacked clarity and specific descriptions [5]. San Diego’s submission earned a higher score (3.5) because of its comprehensive guidance, documentation requirements, code references, and electronic submission instructions [4].

## Scoring for Southington, Connecticut

In the “Process Description” category, Southington was acknowledged for its permitting document, which is specifically designed for EV charging station installation projects, and for describing the entire procedure, from application to final inspection. Moreover, the AHJs were credited for outlining the diverse permit types necessary for EV charging station installation projects, and for differentiating between residential and commercial uses. According to Figure S3, the document specifies that residential applications need only an electrical permit, whereas commercial applications necessitate both electrical and building permits. This differentiation enhances clarity, which ultimately streamlines the applicant’s process. However, the process description provided by Southington was missing several key components, which had a negative impact on the overall score. The application lacked explicit, sequential guidance; a checklist of required permits; clarification regarding processing durations; and details concerning associated fees. Moreover, the documentation failed to include exceptions or expedited review criteria, nor did it specify eligible applicant types. Although the lack of these features does not inherently indicate a deficient permitting process, specifying their availability could enhance the application process’s clarity and utility for users.

Within the “Requirement Explanations” category, Southington was credited for specifying the requisite documents for permit applications and for clarifying which documents pertain to residential versus commercial EV charging station installation projects, as evidenced in Figure S3. Despite this, the AHJ did not meet the remaining requirements in this category. In particular, the document omitted references to the current model codes implemented by the Authority Having Jurisdiction (AHJ), did not explain formatting stipulations for the documentation outlined in Table 3 of the manuscript, and did not specify the applicable building or electrical codes for each listed requirement. Furthermore, the AHJ did not address accessibility requirements, nor did it address the specifications for various charger types (Level 1, Level 2, or DCFC). Without these details, the applicant must either find the information on their own or ask whether other requirements apply. The efficiency of the permit application process would be improved by providing this information in advance.

Figure S3. Section of Southington, Connecticut's EV Charger Permit Requirements document distinguishing requirements for residential versus commercial permit applications.

In the “Technology and Tools” section, the statement by Southington, “*All forms are to be uploaded to the online application*,” helped achieve a higher overall score. The adoption of an online submission system notably increases the permitting process’s efficiency and user-friendliness. However, the AHJ provided no instructions on how applicants may access the online application, nor did it outline the required steps for its completion and submission. Including this information will help streamline the procedure and mitigate applicant confusion. Additionally, the AHJ’s lack of clarification regarding electronic signatures could further facilitate submission procedures.

In the final category, “Communication and Support,” Southington was credited for incorporating permit reviewer contact information within the permitting requirements document. The AHJ furnished the name, email, and telephone number of the building official, as well as contact information for the building department, fire department, and zoning department. The accessibility of contact details negates the requirement for applicants to seek out this information, thereby averting instances of misdirected communication, leading to time savings and enhanced efficiency in the permitting process.

## Scoring for San Diego, California

San Diego demonstrated exceptional performance in the “Process Description” category, satisfying almost every criterion and accumulating points for each requirement met. The AHJ provides instructions for permit applications on a dedicated webpage bulletin, titled “How to Obtain a Permit for Electric Vehicle Charging Systems”. The website furnishes a thorough overview of the EV charging permitting procedure, encompassing application and instructions. The information bulletin is well-organized, with a table of contents located at the top of the webpage, linking to the subsequent sections: Permit Requirements, Submittal Requirements, Future Installations, Permit Application Types, Fees, Inspections, References, Checklist, and Previous Versions. The webpage additionally provides a comprehensive checklist of permit stipulations and outlines necessary documentation, including content guidelines. District requirements for various site types, including single-family residential, multi-family residential, commercial, and other non-residential uses, are further outlined in the bulletin.

It distinctly outlines necessary permits, required documentation, applicable fees, and potential exemptions for each site type. The description of the procedure is noteworthy for its meticulousness and straightforwardness. However, a significant limitation of this page is the absence of information on the permitting process’s timelines, which would improve applicants’ ability to expect outcomes. A significant factor in San Diego’s high score in the “Requirement Explanations” category was the permitting webpage's detailed outline of required documents, engineering drawing plans, and other documentation.

The AHJ comprehensively explained the required details for each component of the engineering plans. The site plan document's requirements, for instance, specify details such as “existing buildings and structures,” “existing parking spaces and proposed EV charging station parking locations,” and “the dimensioned layout of current accessible parking spaces” including access aisles,” among other explicit information criteria. Explicit information requirements result in compliant engineering plans, thereby minimizing the need for revisions from incomplete data. Additionally, the AHJ included links to the referenced codes and the specific relevant electrical or building code article and section number for each requirement, enhancing compliance by eliminating ambiguity concerning applicable codes. The permit document also includes accessibility considerations, specifying sites requiring accessible EV charging stations and referencing applicable accessibility code requirements. It would be beneficial to include visual aids to illustrate the layout specifications for accessible EV parking spaces in accordance with regulations. In addition, the process description does not indicate whether requirements vary by charging type (Level 1, Level 2, and DCFC). While it may seem that the requirements are the same for different charging types, explicitly stating this information would enhance clarity for applicants.

Within the “Technology and Tools” category, San Diego achieved exceptional results by meeting all requirements. Referring to Figure S4, the AHJ offers instructions for using the online permit submission portal, with direct access links and explicit guidance for various site types, including single-family, multi-family, and non-residential.

The addition of these resources facilitates the application process across different project types. Additionally, the authorization of electronic signatures for permitting documents streamlines the procedure, minimizing administrative impediments and improving applicant convenience.

Figure S4. Section of San Diego, CA's permitting document on how to obtain a permit for electric vehicle charging systems. This section outlines which permit application types to select in the online submission portal according to site type.

While San Diego's performance was satisfactory in other areas, it failed to satisfy the

“Communication and Support” requirements. The webpage does not provide clear contact details or instructions on how to reach appropriate city officials regarding permit application inquiries or updates.

# References

1.	Buster, G. Energy Language Model (ELM): Ordinance_GPT. https://github.com/NREL/elm/tree/main/examples/ordinance_gpt (2024).

2.	Buster, G. *et al.* Supporting Energy Policy Research with Large Language Models. *Energy AI* **18**, 100431 http://arxiv.org/abs/2403.12924 (2024).

3.	Renganathan, U., Olson, R., Desai, R. R. & Buster, G. LLMs for EV Infrastructure Permitting. https://data.openei.org/submissions/8540 (2025).

4.	San Diego Development Services. How to Obtain a Permit for Electric Vehicle Charging Systems. https://www.sandiego.gov/sites/default/files/dsdib187.pdf (2025).

5.	Weichsel, J. *EV Charger Permit Requirements*. https://cms9files.revize.com/southingtonct/EV%20Charger%20Handout%2010.1.22.pdf (2022).
